# Optimal organ-sparing intensity-modulated radiation therapy (IMRT) regimen for the treatment of locally advanced anal canal carcinoma: a comparison of conventional and IMRT plans

**DOI:** 10.1186/1748-717X-2-41

**Published:** 2007-11-15

**Authors:** Cathy Menkarios, David Azria, Benoit Laliberté, Carmen Llacer Moscardo, Sophie Gourgou, Claire Lemanski, Jean-Bernard Dubois, Norbert Aillères, Pascal Fenoglietto

**Affiliations:** 1 Département de Radio-Oncologie, Hôpital Maisonneuve-Rosemont, Montréal, Canada.; 2 Département d'Oncologie Radiothérapie et de Radiophysique, CRLC Val d'Aurelle-Paul Lamarque, Montpellier, France.; 3 Unité de Biostatistiques, CRLC Val d'Aurelle-Paul Lamarque, Montpellier, France.

## Abstract

**Background:**

To compare the dosimetric advantage of three different intensity-modulated radiation therapy (IMRT) plans to a three dimensional (3D) conventional radiation treatment for anal cancer with regards to organs-at-risk (OAR) avoidance, including iliac bone marrow.

**Methods:**

Five patients with T1-3 N0-1 anal cancer and five with T4 and/or N2-3 tumors were selected. Clinical tumor volume (CTV) included tumor, anal canal and inguinal, peri-rectal, and internal/external iliac nodes (plus pre-sacral nodes for T4/N2-3 tumors). Four plans were generated: (A) AP/PA with 3D conformal boost, (B) pelvic IMRT with conformal boost (C) pelvic IMRT with IMRT boost and (D) IMRT with simultaneous integrated boost (SIB). The dose for plans (A) to (C) was 45 Gy/25 followed by a 14.4 Gy/8 boost, and the total dose for plan (D) (SIB) was 59.4 Gy/33. Coverage of both PTV and the volume of OAR (small bowel, genitalia, iliac crest and femoral heads) receiving more than 10, 20, 30, and 40 Gy (V10, V20, V30, V40) were compared using non parametric statistics.

**Results:**

Compared to plan (A), IMRT plans (B) to (D) significantly reduced the V30 and V40 of small bowel, bladder and genitalia for all patients. The V10 and V20 of iliac crests were similar for the N0-1 group but were significantly reduced with IMRT for the N2-3/T4 group (V20 for A = 50.2% compared to B = 33%, C = 32.8%, D = 34.3%). There was no statistical difference between 2-phase (arm C) and single-phase (SIB, arm D) IMRT plans.

**Conclusion:**

IMRT is superior to 3D conformal radiation treatment for anal carcinoma with respect to OAR sparing, including bone marrow sparing.

## Background

The standard of care for the curative treatment of anal canal carcinoma has evolved over the past three decades, from abdomino-perineal resection and life-long colostomy to organ preservation therapy using combined radiation and chemotherapy. Organ preservation is now achieved in more than 70% of cases [1-7]. The downfall of this approach is significant acute toxicity in the form of moist desquamation, genitourinary and gastrointestinal effects, and hematologic compromise. In turn, this can lead to undue treatment breaks and long overall treatment times that may negatively influence outcome [8,9]. To compound the difficulty of decreasing side-effects, retrospective data also seems to indicate a positive impact on local control of dose escalation to or above 54 Gy [10-12]. While the high acute toxicity associated with this treatment might be reduced by an alternate chemotherapy regimen, this approach remains investigational.

In other cancers faced with similar difficulty, it has been shown that side effects may be reduced by using more conformal therapy in the form of intensity-modulated radiation therapy (IMRT) [13-20]. IMRT is well suited for anal cancer because of the many critical structures adjacent to the target volume, which receive significant doses with conventional (AP-PA) field arrangements or 3D conformal treatment. This approach has already been investigated at the University of Chicago [21] where 17 patients were treated with IMRT, thereby reducing the mean and threshold doses to the small bowel, bladder, and genitalia/perineum compared to conventional treatment. There were no delays attributable to gastrointestinal or skin toxicity. However, slightly worse hematologic toxicity (53% grade 3–4 acute blood/bone marrow toxicity) were reported compared to other studies. It was hypothesized that this was the result of higher radiation threshold doses to iliac bone where most of the adult bone marrow reserve lies. The IMRT optimization had taken into account the iliac bone as a posterior "blocking" structure but without specific dose constraints.

Considering these results, our goal was to find an optimal radiation protocol which can treat anal cancer at high doses while providing not only gastrointestinal and dermatologic but also adequate iliac bone marrow sparing. We designed a study that compares three different IMRT plans delivering 59.4 Gy in 33 fractions for each of 10 patients to a conventional three-dimensional AP-PA plan followed by a 3-dimensional conformal radiotherapy (3D CRT) boost (plan A). The three IMRT plans were: a pelvic IMRT plan followed by a 3D CRT boost (plan B), a pelvic IMRT plan followed by a sequential IMRT boost (plan C) and finally, an IMRT plan with simultaneous integrated boost (SIB, plan D). Our findings are presented here along with a review of pertinent IMRT planning studies of anal cancer.

## Methods

### Simulation, target contouring and fractionation

Ten patients previously treated with curative intent chemoradiation at the Val d'Aurelle-Paul Lamarque Cancer Institute (Montpellier, France) during the last 2 years were selected. Criteria for inclusion were that patients must have had complete imaging of iliac crests on the planning simulation scan to permit dose-volume histogram (DVH) comparisons of iliac bone marrow. Five patients were staged T1-3 N0-1 and the remaining five were T4 and/or N2-3 tumors according to the 6^th ^edition of the American Joint Committee on Cancer staging manual. Details of staging are shown in Table 1.

**Table 1:** Patient tumor staging

N0–N1 group					N2–N3/T4 group				
No. 1	No. 2	No. 3	No. 4	No. 5	No. 6	No. 7	No. 8	No. 9	No. 10

T2 N0	T3 N0	T3 N0	T2 N1	T3 N1	T4 N2	T1 N2	T4 N1	T3 N2	T4 N2

Patients were simulated in the supine position without a custom immobilization device using a planning CT scan (PQ 2000 CT Simulator, Marconi Medical Systems, Cleveland, OH) with 4 mm thick slices. Intravenous contrast and an anal marker were recommended but were not compulsory.

Relevant structures were manually contoured on each axial CT scan slice by a single radiation oncologist. The gross tumor volume (GTV) was contoured based on findings from physical examination, diagnostic CT scan, MRI and endoscopic ultrasound in all patients. The clinical target volume 1 (CTV1) consisted of the GTV expanded by a three-dimensional 1 cm margin, the anal canal, and the draining lymphatic regions, including the perirectal, internal iliac, external iliac, obturator and inguinal lymphatics in all cases. For the five patients with N2–3 and/or T4 tumors, the pre-sacral nodes were also included. These nodal regions were defined by encompassing the contrast enhanced vessels with a 1 cm margin. The perirectal region was defined as the rectal wall and the fat containing mesorectum. Finally, the CTV1 was uniformly expanded by 1 cm to produce the planning target volume 1 (PTV1). The boost volume (PTV2) consisted of the pre-treatment GTV expanded radially by 1.5 cm. OAR included the small bowel, bladder, perineum and external genitalia (penis and scrotum in men and vulva in women), iliac crests (from bony top to the superior part of acetabulum inferiorly) and femoral heads. No additional margin around OAR was used to account for possible inter or intra fraction motion. Depending on patient anatomy, anterior and/or posterior blocking structures could be used in the IMRT planning process. Dose distributions did not take into account corrections for tissue heterogeneities.

### Conventional three-dimensional planning

Arm A consisted of conventional three-dimensional AP/PA fields plans to 45 Gy in 25 fractions in phase 1 followed by a 3D conformal 3-field boost to 59.4 Gy. Using the PTV described above and taking into account the beam penumbra, the resulting field borders for the AP and PA fields were: upper limit at the level of S2–S3 for N0-1 tumors and at the level of L5-S1 for N2-3 and T4 tumors, lower border at 2.8–3 cm below the tumor. The lateral limits where set at the antero-superior iliac spine. Multi-leaf collimators were used to adjust dose conformation around the PTV. Six and 18 MV photons were used for the AP and PA fields, respectively. The radiation dose was prescribed to the PTV, such that 100% of the PTV received > 95% of the prescribed dose and that no region in the field received greater than 107% of the prescribed dose. Variable weighting of the fields and wedges were used to optimize the plan and improve dose homogeneity. Direct electron beams could be used to supplement the dose to the inguinal regions as needed.

### IMRT planning

Arms B, C and D used IMRT plans generated by a commercial inverse planning software (Eclipse, Helios, version 7.2.34, Varian, Palo Alto, CA) and sliding window technique. Beam geometry consisted of seven non-coplanar fields for the whole pelvis (phase 1) with the following gantry angles: 0°, 45°, 110°, 165°, 195°, 250° and 325°. A 5-field technique was used for the IMRT boost of arm C (45°, 110°, 180°, 250° and 315°). Patients were treated with an 18 MV linear accelerator with dynamic multileaf collimator (21 EX, Varian, Palo Alto, CA). This energy was chosen for IMRT because the resulting plans have less complex fluence maps and require fewer monitor units as compared to those generated with 6 MV photons.

Arm B consisted of pelvic IMRT delivering 45 Gy in 25 fractions to PTV1 in phase 1 followed by a conformal 5-field radiotherapy boost to PTV2 for a total dose of 59.4 Gy in 33 fractions. Arm C consisted of the same pelvic IMRT phase 1 of 45 Gy as in Arm B but the subsequent boost to PTV2 was delivered with IMRT, for a total dose of 59.4 Gy in 33 fractions. Finally, arm D consisted of pelvic IMRT with a SIB delivering 49.5 Gy in 33 fractions (biological equivalent dose of 45 Gy in 25 fractions for early response tissues) to PTV1 and 59.4 Gy in 33 fractions to PTV2.

The PTV and OAR optimization constraints were iteratively adjusted in Helios until a clinically acceptable treatment plan was obtained. Typical optimization parameters of OAR for IMRT planning are shown in Table 2.

**Table 2:** Typical OAR optimization parameters for IMRT

Organ	Threshold dose (Gy)	Volume above limit (%)
Bladder	30	80
	40	40
	0	50

Small Bowel	30	40
	40	30
	0	50

Genitalia/perineum	30	35–45
	40	5–10
	0	48

Iliac crests	10	35–45
	20	25–30
	0	50

Femoral heads	45	5
	0	55

Dose-volume histograms (DVH) were generated for all of these structures. Treatment plans were evaluated using visual inspection and evaluation of dose distributions on the CT-slices as well as DVH analysis. For a plan to be considered clinically acceptable, 95% of the PTV must have received ≥ 95% of the prescribed dose, no region could receive more than 65 Gy (109% of the prescribed dose) and doses to OAR were minimized.

### Statistical Analysis

The mean percentage volume of bladder, genitalia/perineum, and small bowel receiving more than 30 and 40 Gy (V30 and V40) were calculated from the plans, as well as the mean percentage volume of iliac crests receiving more than 10 and 20 Gy (V10 and V20). A two sample Wilcoxon rank-sum (Mann-Whitney) test was used for all comparisons between treatment techniques. The three planning techniques incorporating IMRT (arms B, C, and D) were each compared with the conventional 3D plan (arm A), and then arm C was compared to arm D. A *p *value less than 0.05 was used to indicate statistical significance.

## Results

### Comparison of target volume coverage between IMRT and conventional 3D plans

All treatment plans showed adequate coverage of the target volume, with more than 95% of volume of PTV1 and PTV2 receiving greater than 95% of the prescribed dose. Also, 98% of the target volume received more than 90% of the prescribed dose in all cases. Evaluation of homogeneity using maximum dose to PTV 1 and PTV 2 was lower with the IMRT plans (Arms B to D) than with the conventional 3D plans (Arm A). For arms B to D, the mean volume of PTV receiving more than 107% of the prescribed dose was 0.003% for PTV 1 and 0.016% for PTV 2. Furthermore, no volume received more than 110% for any plan.

**Figure 1:**
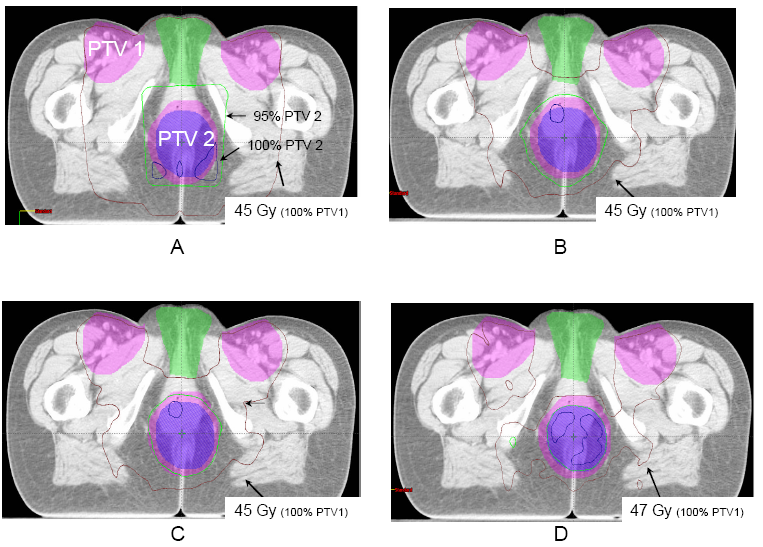
Dose distributions by treatment arm on the same axial CT slice through both target volumes (PTV1 and PTV2) and the external genitalia in a female patient. This CT slice shows the sparing of the genitalia by the 45 Gy isodose curve in arms B, C, and D as compared with arm A.

**Figure 2:**
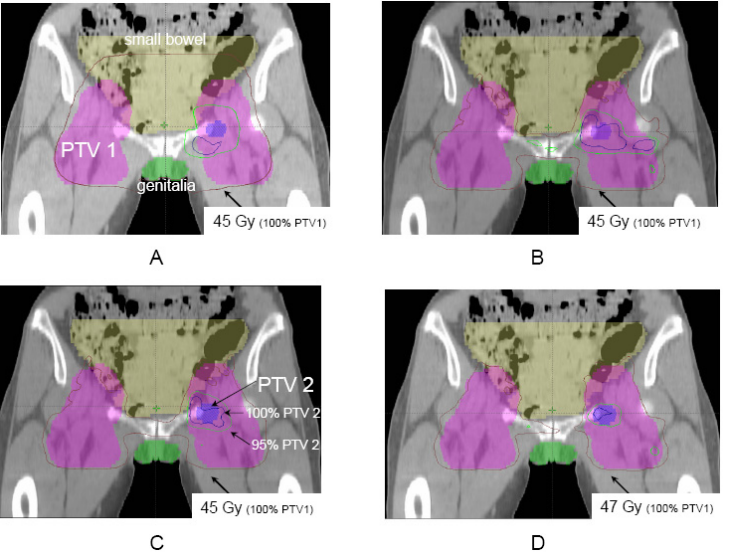
Dose distributions by treatment arm on the same coronal CT slice through both target volumes and avoidance structures. This CT slice also shows the sparing of the external genitalia, small bowel and iliac crests in arms B, C, and D as compared with arm A.

Figure 1 shows typical sparing of the genitalia from the total dose prescribed to PTV1 in the IMRT arms as compared to conventional 3D plans. This is also shown in Figure 2, where sparing of the small bowel can equally be noted.

### Comparison of OAR sparing between IMRT and conventional 3D plans

Incorporation of IMRT in phase 1 or in phase 1 and 2 significantly reduced the volume of bladder and genitalia/perineum receiving ≥ 30 Gy and ≥ 40 Gy for N0-1 and N2-3/T4 patients as compared to conventional 3D plans (Tables 3 and 4). For example, the mean volume of genitalia/perineum receiving ≥ 40 Gy is 7% and 3% for arms C and D compared to 80% with the conventional 3D plan for N0-1 patients (*p *< 0.05). Similarly, for N2-3/T4 patients, 9% and 7% of the genitalia received ≥ 40 Gy for arms C and D compared to 85% with the conventional 3D plan (*p *< 0.05). There is also significantly less small bowel receiving ≥ 30 Gy and ≥ 40 Gy for both patient groups using IMRT, although this did not reach statistical significance for the V30 for the IMRT with SIB (arm D) compared to the conventional 3D treatment (*p *= 0.07 for both patient groups).

**Table 3:** Mean percent volume of OAR receiving various dose levels for N0–N1 patients (upper limit of fields at S2–S3) for a 59.4 Gy treatment

		Mean volume receiving above threshold dose (%) ^†^			
Organ	Threshold dose (Gy)	A	B	C	D

Bladder	30	100	87.8* (80–95)	86.7* (78–92)	80.8* (74–83)
	40	100	65.2* (55–76)	57.4* (43–69)	46.2* (35–55)
Genitalia/perineum	30	85.8 (72–99)	47.6* (23–88)	36.4* (23–52)	26.6* (13–44)
	40	80.4 (65–98)	20.2* (0–54)	7.2* (0.2–18)	2.7* (0–7)
Small bowel	30	59.3 (37–72)	40.1* (28–49)	39.6* (28–48)	39.7 (29–45)
	40	56.1 (35–68)	25.0* (17–31)	24.5* (16–30)	26.7* (19–30)
Iliac crests	10	39.8 (33–52)	37.4 (33–44)	37.1 (33–44)	39.6 (35–47)
	20	33.7 (28–47)	28.4 (26–30)	28.2 (26–30)	28.3 (27–30)
Femoral heads	45	33.8 (25–51)	23.3 (8–45)	14.6* (2–30)	20.9* (12–30)

†Mean in % (range).

* denotes statistically significant *p *values (*p *< 0.05) as compared with arm A.

**Table 4:** Mean percent volume of OAR receiving various dose levels for N2-3 and/or T4 patients (upper limit of fields at L5-S1) for a 59.4 Gy treatment

		Mean volume receiving above threshold dose (%)^†^			
Organ	Threshold dose (Gy)	A	B	C	D

Bladder	30	100	88.6* (76–100)	84.9* (71–99)	77.0* (68–84)
	40	100	60.2* (43–74)	52.5* (39–70)	39.0* (30–54)
Genitalia/perineum	30	87.0 (60–100)	64.4 (26–93)	46.2* (26–68)	32.8* (20–53)
	40	85.1 (56–99)	25.9* (2–43)	9.8* (1–26)	7.4* (0–26)
Small bowel	30	64.4 (48–89)	43.6* (38–52)	43.0* (37–50)	45.8 (39–55)
	40	62.1 (45–87)	26.5* (21–30)	25.8* (19–30)	28.3* (20–35)
Iliac crests	10	59.9 (57–63)	52.2* (45–57)	51.4* (45–56)	56.0 (47–60)
	20	50.2 (47–54)	33.0* (30–37)	32.8* (30–37)	34.3* (30–38)
Femoral heads	45	29.9 (21–39)	15.7* (8–21)	8.8* (6–14)	14.6* (11–17)

†Mean in % (range).

* denotes statistically significant *p *values (*p *< 0.05) as compared with arm A.

The results are shown separately in Table 3 for the N0-1 patients and Table 4 for N2-3/T4 patients since the upper field limit was different for the two groups which influences the dose to OAR, particularly to the iliac crests and small bowel.

Also, since high doses to even a small volume of small bowel may be thought to be deleterious, we computed the maximum dose to 1% (D1) of the small bowel (Table 5). Interestingly, patient n°8 would have received close to the prescribed dose if treated with arm A. He presented a large primary tumor which extended superiorly into the rectum, invaded the prostate and lead to a recto-vesical fistula. For this patient, the D1 for arms C and D were 52 and 49.5 Gy, respectively, which compare favorably to the 58.5 Gy of the conventional 3D plan (arm A).

**Table 5:** Dose received (Gy) by 1% of the small bowel (D1)

	volume (cc)	A	B	C	D
No. 1(T2 N0)	599	51.5	46	46	47.5
No. 2 (T3 N0)	631	47	45.5	45	50
No. 3 (T3 N0)	670	51.5	48	47	49
No. 4 (T3 N1)	1434	48	47.5	47	49
No. 5 (T3 N1)	759	54.5	53.5	51.5	49.5
No. 6 (T4 N2)	1080	54	52.5	52	49.5
No. 7 (T1 N2)	2046	51.5	45.5	44.5	48.5
No. 8 (T4 N1)	859	58.5	56.5	52	49.5
No. 9 (T3 N2)	938	48	48	47.5	49
No. 10 (T4 N2)	1252	49	47.5	47.5	50

The mean volume of iliac crests receiving ≥ 10 Gy and ≥ 20 Gy was similar between the four treatment arms for N0-1 patients, ranging from 37 to 40% for the V10 and 28 to 34% for the V20. However, for N2-3 and/or T4 patients with an upper field limit at L5-S1, significant iliac bone marrow sparing was found with all but one (V10 with arm D) treatment arms utilizing IMRT compared to the conventional 3D treatment. The absolute BMS gain for the V20 is between 15 and 17%, with a V20 of 50.2% for arm A compared to 33, 32.8, and 34.3% for arms B, C, and D, respectively (all *p *< 0.05).

The volume of femoral heads receiving ≥ 45 Gy was reduced with all plans incorporating IMRT compared to the conventional 3D plan for both patient groups and the mean dose to the femoral heads was similar in all treatment plans.

**Figure 3:**
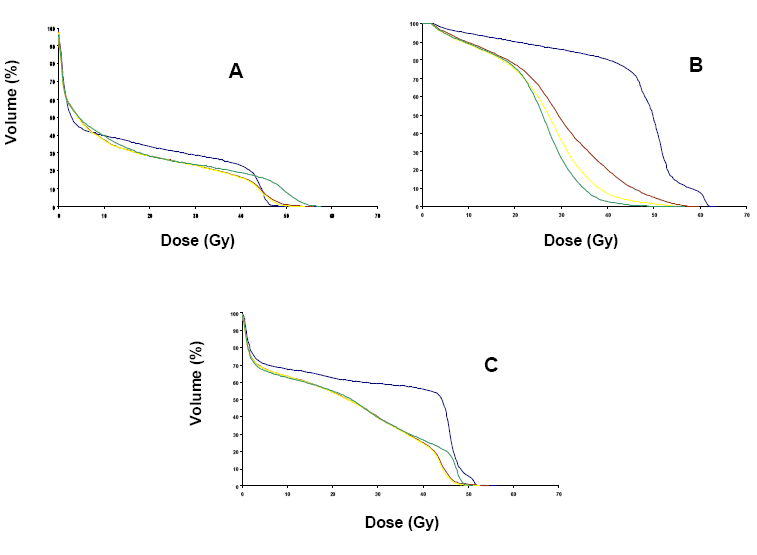
(3A), Mean Dose-Volume Histograms (DVH) for iliac crests of N0-1 group. Arm A, blue; arm B, brown; arm C, yellow; arm D, green; (3B), Mean Dose-Volume Histograms (DVH) for genitalia/perineum of N0-1 group. Arm A, blue; arm B, brown; arm C, yellow; arm D, green; (3C), Mean Dose-Volume Histograms (DVH) for small bowel of N0-1 group. Arm A, blue; arm B, brown; arm C, yellow; arm D, green.

**Figure 4:**
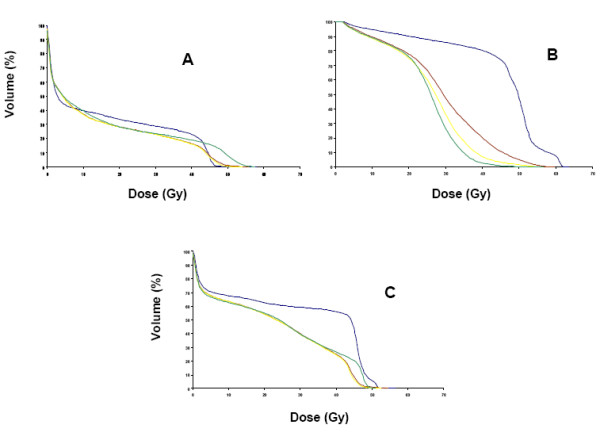
(4A), Mean Dose-Volume Histograms (DVH) for iliac crests of N2-3/T4 group. Arm A, blue; arm B, brown; arm C, yellow; arm D, green; (4B), Mean Dose-Volume Histograms (DVH) for genitalia/perineum of N2-3/T4 group. Arm A, blue; arm B, brown; arm C, yellow; arm D, green; (4C), Mean Dose-Volume Histograms (DVH) for small bowel of N2-3/T4 group. Arm A, blue; arm B, brown; arm C, yellow; arm D, green.

Figures 3 and 4 depict mean DVHs by treatment arm for OAR according to treatment groups.

### Additional comparison between IMRT plans

We carried out comparisons of all evaluated endpoints between the two treatments arms consisting solely of IMRT (arms C and D). We failed to find any significant statistical differences between these two treatment plans with respect to target volume coverage and critical organ sparing.

## Discussion

Concurrent chemoradiation is the established standard of care for locally advanced carcinoma of the anal canal. Attempts to decrease the important side-effects of this treatment by modifying the chemotherapy regimen with the suppression of Mitomycin-C (MMC) have resulted in increased loco-regional recurrence and higher colostomy rates [5,7]. Furthermore, a recent Intergroup trial led by the RTOG to evaluate the possible replacement of MMC by induction and concomitant cisplatin (CDDP) has shown disappointing preliminary results, with a non-statistically significant higher colostomy rate in the CDDP arm, which was however better tolerated in terms of hematologic toxicity [22]. While longer follow-up is needed, at present MMC still plays a major role in the management of carcinoma of the anal canal.

As well, decreasing the radiation dose or using split-course techniques is no longer recommended. Instead, several groups have focused their attention on modifying the radiation delivery technique using a 3D-CRT "diamond technique" [23,24] or through IMRT [21,25,26]. While IMRT has successfully reduced small bowel, perineal and genitalia doses, hematologic toxicity remains a clinical concern. This is in part because a large proportion of the body bone marrow reserve is located within the lumbar spine and pelvic bones [27]. Moreover, apart form the ovaries, the bone marrow is the most radiosensitive pelvic tissue [28], and concurrent chemotherapy likely lowers this threshold dose and is in itself myelotoxic.

The issue of chemotherapy-induced bone marrow suppression worsened by pelvic irradiation has been studied in patients with gynecologic malignancies. Brixey *et al*. [20] showed that intensity-modulated whole pelvic radiotherapy (IM-WPRT) decreased the number of women experiencing Grade 2 or greater white blood cell count (WBC) toxicity as compared with conventional WPRT in 36 patients receiving chemotherapy. This resulted in chemotherapy being held back less often for hematologic toxicity. Of note, these benefits in hematologic toxicity were initially seen without explicitly using a bone marrow sparing (BMS) radiation technique. Subsequently, the same group used BMS IM-WPRT and significantly reduced the volume of bone marrow receiving > 18 Gy compared with both IM-WPRT and 4 field box techniques [29].

In the current trial, we compared four radiation delivery techniques with the intent of determining the optimal regimen that achieves maximal target volume coverage and provides adequate gastrointestinal and dermatologic sparing without compromising the bone marrow. We have shown that using BMS IM-WPRT throughout the entire treatment (arms C and D) is feasible and reduces threshold radiation doses to the small bowel, bladder, genitalia and femoral heads as compared to conventional AP/PA plans with 3D conformal boost. The mean values that we obtained for these critical structures with a prescription dose of a 59.4-Gy treatment are comparable to those obtained by Milano *et al*. [21] in their IMRT treatment arm with a dose of 45 Gy. This was achieved in our study with both N0–N1 tumors with an upper field border at the level of S2–S3 and in N2–N3/T4 tumors with an upper field border at the level of L5-S1 that allowed proper coverage of the mesorectal and presacral nodes.

Furthermore, BMS WP-IMRT provided comparable mean and threshold doses to the iliac crests in N0–N1 tumors, but statistically reduced mean and threshold doses to this structure in N2-3 and/or T4 tumors. The mean V10 and V20 for our N2-3/T4 tumor patient subgroup were 53 and 33%, respectively, compared to the 73% and 59% obtained by Milano *et al*. [21]. Whether these lower doses to iliac bone marrow will result in less acute hematologic toxicity remains to be seen in further clinical studies.

The dose given to femoral heads in our trial deserves more discussion. Although the volume of femoral heads receiving ≥ 45 Gy was reduced with all plans incorporating IMRT compared to the conventional 3D plan, it was higher than expected. In comparison, Hsu et al. [26] treated five patients with T2 N0-1 tumors with definitive chemo-radiation using IMRT plans. For each patient, AP/PA plans with supplemental inguinal electrons boosts, 4-field box, 7-field IMRT, and 7-field IMRT integrated boost plans were generated. The volume of bowel and bladder receiving threshold doses were similar to ours, but the V45 of the femoral heads is 0% in both of their IMRT arms, which is much less than what we obtained. This may be explained by several factors including different definitions of target volumes, more stringent dose constraints used in their study (i.e. femoral head V45 < 1%) and the smaller tumor sizes of their patients which were all staged T2 N0-1. Furthermore, the mean V20 for the iliac crests was 77% for both their IMRT arms as compared with 28% and 33% in our N0-1 and N2-3/T4 groups, respectively. Interestingly, Milano *et al*. [21] noted higher than expected hematologic toxicity with an iliac crest V20 of 59% and V10 of 73%. It is possible that sparing of the femoral heads and/or other organs comes at the cost of increasing dose to the iliac crests. The results of our study reinforce the idea that specific dose constraints for all OAR must be considered during the IMRT optimization process in anal cancer, namely with regards to the iliac crests.

Another study which focused on femoral head dose was published by Chen *et al*. [25]. They compared 7 coplanar fields IMRT with conventional plans for the coverage of pelvic and inguinal/femoral nodes in two patients with anal cancer. The whole pelvic dose was 36 Gy in 20 fractions. The mean dose to the femoral head was 58.3% and 59.5% of the prescription dose for their 2 patients using conformal avoidance IMRT. Similarly, the mean dose to the femoral head with the IMRT plans in our study ranged from 52.2% to 56.5% of the prescribed dose. This confirms that the whole pelvis may be treated to 45 Gy with an additional 14.4 Gy boost to the tumor while keeping the mean dose to the femoral heads at a relatively constant percentage of the prescription dose.

A third IMRT study using a single-phase dose painting technique has been described by the Boston Medical Center and Massachusetts General Hospital. Six patients were treated (in text, RTOG 0529 protocol draft, p.11) and dose-painting IMRT provided better normal tissue sparing than 3D CT-based conformal therapy plans. No patient required a treatment break of more than one week and all patients completed therapy as initially planned. Comparisons with our dosimetric study are limited since they did not use the same threshold OAR doses. However, the mean V30 and V40 values for the bladder were similar to ours, whereas our mean V30 values for the genitalia were similar or lower than the mean V35 that they obtained. However, they achieved lower V30 and V40 values for the small bowel and much lower V45 for the femoral heads. Differences may be due to the lower total dose, which ranged from 42 to 45 Gy to elective nodes and from 50.4 to 54 Gy to gross tumor. The RTOG is currently accruing patients for a phase II trial of dose-painted IMRT.

There was no significant difference between the 2-phase (arm C) and single-phase IMRT plans (arm D). Both of these arms seem superior, at least for genitalia sparing, to arm B although there was no statistical analysis comparing these treatment arms. As for the greater volume of iliac crest receiving doses above 45 Gy observed in the DVH of arm D (Fig. 3), this difference is not clinically significant since it is probable that theses segments of marrow are not functional after having received 45 Gy. Also, the doses shown in the DVH are physical doses. If we consider the radiobiological equivalent dose calculated in daily 1.8 Gy fractions for the SIB arm (arm D), the DVH will be shifted to the left and 49.5 Gy in 1.5 Gy fractions would be approximately equivalent to 45 Gy in 1.8 Gy fractions.

## Conclusion

BMS-IMRT in the treatment of anal canal cancer reduces dose to surrounding normal structures compared with conventional 3D AP/PA planning followed by a conformal boost. Other single institution studies have shown that this treatment is well tolerated and decreases acute toxicity and treatment breaks. Longer follow-up is needed to determine if late toxicity will be reduced and if loco-regional control and survival will be equivalent. In our center, we have determined that a single-phase IMRT treatment with simultaneous integrated boost is dosimetrically equivalent and more convenient than two-phase treatment techniques since there is only one optimization and quality assurance session by the physicist. This approach is currently being evaluated in phase II protocols by our institution and by the RTOG. A more standardized approach to target volume definition and dose prescription for IMRT of the anal canal is warranted.

## List of abbrevations

3D: three dimensional

3D CRT: 3-dimensional conformal radiotherapy

BMS: bone marrow sparing

CDDP: cisplatin

CTV: clinical target volume

DVH: dose-volume histogram

GTV: gross tumor volume

IMRT: intensity modulated radiotherapy

IM-WPRT: intensity-modulated whole pelvic radiotherapy

MMC: Mitomycin-C

OAR: organ at risk

PTV: planning target volume

SIB: simultaneous integrated boost

V10, V20, V30, V40: volume of organ at risk receiving more than 10, 20, 30, and 40 Gy

WBC: white blood cell count

WPRT: whole pelvic radiotherapy

## Competing interests

The author(s) declare that they have no competing interests.

## Authors' contributions

CM and PF conceived the study, collected data, and drafted the manuscript.

BL, CLM, JBD, CL, and DA participated in coordination and helped to draft the manuscript.

SG performed the statistical analyses.

DA provided mentorship and edited the manuscript.

All authors have read and approved the final manuscript.
